# Role of liver progenitors in acute liver injury

**DOI:** 10.3389/fphys.2013.00258

**Published:** 2013-09-26

**Authors:** Jan Best, Laurent Dollé, Paul Manka, Jason Coombes, Leo A. van Grunsven, Wing-Kin Syn

**Affiliations:** ^1^Department of Gastroenterology and Hepatology, University Hospital EssenEssen, Germany; ^2^Liver Cell Biology Lab (LIVR), Department of Cell Biology (CYTO), Faculty of Medicine and Pharmacy, Vrije Universiteit BrusselBrussels, Belgium; ^3^Regeneration and Repair Group, Foundation for Liver Research, The Institute of HepatologyLondon, UK

**Keywords:** hepatic failure, acute liver failure, severe acute liver injury, liver progenitor cells, oval cells, stem cells, liver regeneration

## Abstract

Acute liver failure (ALF) results from the acute and rapid loss of hepatocyte function and frequently exhibits a fulminant course, characterized by high mortality in the absence of immediate state-of-the-art intensive care and/or emergency liver transplantation (ELT). The role of hepatocyte-mediated liver regeneration during acute and chronic liver injury has been extensively investigated, and recent studies suggest that hepatocytes are not exclusively responsible for the regeneration of the injured liver during fulminant liver injury. Liver progenitor cells (LPC) (or resident liver stem cells) are quiescent in the healthy liver, but may be activated under conditions where the regenerative capacity of mature hepatocytes is severely impaired. This review aims to provide an overview of the role of the LPC population during ALF, and the role of putative cytokines, growth factors, mitogens, and hormones in the LPC response. We will highlight the potential interaction among cellular compartments during ALF, and discuss the possible prognostic value of the LPC response on ALF outcomes.

## Introduction

Acute liver failure (ALF) is a rare clinical syndrome that affects around 2000 individuals in the USA annually (Polson and Lee, 2005) and frequently exhibits a fulminant course, characterized by high mortality in the absence of immediate state-of-the-art intensive care and/or emergency liver transplantation (ELT).

ALF results from the acute and rapid loss of hepatocyte function, and is associated with coagulopathy [International Normalized Ratio (INR) >1.5] and hepatic encephalopathy (HE) in a patient without pre-existing liver disease. Typically, the time from onset of symptoms to development of HE is up to 8 weeks, but may take up to 26 weeks (Polson and Lee, 2005). Individuals who develop hepatic dysfunction (i.e., coagulopathy), in the absence of HE are defined as having severe acute liver injury (sALI). ALF can occur as a result of various etiologies. In a German study, triggers such as non-acetaminophen drug-induction (including idiosyncratic toxic reactions, e.g., Phenprocoumon) (32%), indeterminate or sero-negative hepatitis (24%), viral hepatitis (such as hepatitis A, B, E) (21%) (Jochum et al., 2009), and acetaminophen overdose (9%) (Hadem et al., 2012) appear to be most frequent causes of ALF. Other etiologies include autoimmune disease (or hepatitis) (Czaja, 2013), ischemia (Henrion, 2012) pregnancy (Ichai and Saliba, 2009), Wilsons disease (Okada et al., 2010), and congestive heart failure (Saner et al., 2009).

The prognosis of ALF is primarily dependent upon the underlying etiology. During ALF, viral-mediated (i.e., direct cytopathic effects), cytokine and/or immune-mediated (i.e., indirect cytopathic effects) hepatocyte necrosis, and apoptosis occur. A regenerative process is triggered, and replication of the remaining healthy hepatocytes ensues, in an attempt to restore hepatic architecture and function. This process is initiated or regulated, at least in part, by three major factors which include cytokines, growth factors, and metabolic signaling pathways. During the early stages of liver damage, inflammatory cytokines trigger healthy hepatocytes to enter the cell cycle. If hepatocyte replication is hampered by excessive parenchymal damage (as generally observed in ALF), or hepatocyte senescence (as occurring in steatotic livers or livers with concomitant chronic injury), resident liver progenitor cells (LPCs) are activated to support, or take over the role of regeneration. However, for many with ALF, this regenerative process is inadequate to match the rapid, confluent loss of hepatocyte mass and function, and liver transplantation offers the only potential hope for survival. Further studies will be needed to ascertain if an enhanced liver progenitor response could lead to better patient outcomes.

A proportion of individuals will recover spontaneously from ALF, and they exemplify the unique capacity of the liver to regenerate completely after injury. Currently used ALF scores/criteria such as the King's College criteria (KCC), Model of end stage liver disease score (MELD), and Bilirubin-lactate-etiology score (BiLE) (Hadem et al., 2008) utilize clinical parameters at the time of admission and/or during the course of ALF, and reliably predict death, but are poor at predicting survival. Recent studies suggest that cell death markers (M65)-based MELD may improve prediction of spontaneous survival (Bechmann et al., 2008, 2010). Hepatocyte cell death is intricately linked to LPC response in chronic liver disease (Jung et al., 2012; Sancho-Bru et al., 2012), hence, raising the possibility that the amount or type of LPC activation (i.e., progenitor cell response) during ALF could also predict ALF outcomes.

The aims of the review are: (A) To provide an overview of the mechanisms of LPC activation and to highlight potential therapeutic targets/strategies in context of ALF, and (B) To discuss the clinical relationship between LPC activation and acute liver damage, and the possible role of LPC activation in predicting ALF outcomes.

## Different cell populations are activated during acute and chronic liver damage

ALF (fulminant hepatic failure) occurs when there is rapid, massive hepatocyte cell death, which leads to significant impairment of liver function. On the other hand, chronic liver injury is driven by progressive hepatocyte injury and death that spans months, years and even decades.

The cellular response that occurs during hepatic injury essentially mirrors the clinical scenario, and as such, is strongly associated with the etiology and severity of injury. Mature hepatocytes constitute the majority cell type in the liver, and are unipotent cells that contribute to normal cell turnover and are able to respond rapidly to injurious stimuli (such as liver resection in man, or partial hepatectomy in mice). In contrast, the LPC compartment is triggered to expand when hepatocyte loss occurs in the presence of residual hepatocyte senescence (i.e., replicative senescence), a feature common to chronic liver disease (Santoni-Rugiu et al., 2005; Dollé et al., 2010). Upon transit amplification, the LPCs infiltrate along the liver plate toward the central vein, and differentiate into hepatocytes to restore liver function and cell mass (Espanol-Suner et al., 2012).

The role of LPC in acute injury or ALF remains poorly defined (Theise et al., 2013). Nevertheless, we and others have recently reported that LPC expansion occurs in mice and humans during acute hepatic injury (or hepatic failure), and propose that the LPC compartment is an important contributor to the restoration of liver parenchyma or function in mice (Ochoa et al., 2010; Khuu et al., 2013). Inhibiting the LPC response in mice after 70% partial hepatectomy led to impaired liver regeneration, as assessed by the liver to body weight ratios, and reduced overall survival. It is likely, however, that the LPC compartment is only activated when there is an insufficient number of healthy residual hepatocytes to undertake the regenerative process. Indeed, Katoonizadeh et al. suggest, that a minimum 50% hepatocyte loss and presence of hepatocyte replicative senescence are necessary triggers for LPC activation (Katoonizadeh et al., 2006).

The activation and expansion of the LPC compartment occurs roughly over 7 days, while process of LPC differentiation into intermediate hepatocytes requires an additional 7 days (Fausto, 2004; Fausto et al., 2006). Thus, the LPC response is a much slower regenerative process (compared with hepatocyte replication), and can be more easily detected in the livers of patients with a sub-acute form of ALF (i.e., such as in those with sero-negative hepatitis). Although there is no direct evidence on the role of LPC in human liver regeneration during ALF, the presence of a ductular response after acute alcoholic hepatitis or ALF, and aggregate data from small animal studies support the hypothesis that activation and differentiation of LPCs might play a pivotal role in regeneration following fulminant hepatocyte loss (Katoonizadeh et al., 2006; Sancho-Bru et al., 2012).

## Signaling pathways and markers of LPC

Under normal circumstances (i.e., in a healthy adult liver), the responsibility of regenerating a liver after an acute insult falls upon the residual hepatocytes. Ordinarily, hepatocytes turn over once or twice a year (Fausto et al., 2006). Upon acute injury, complete hepatocyte regeneration can occur after 2–3 cycles of hepatocyte replication (Michalopoulos, 2010). This regenerative process is orchestrated by cross talk between different liver cell compartments, and mediated by multiple cytokines, growth factors, and mitogens.

The regulation of the LPC response is best characterized in humans and animal models of chronic liver disease. Chronic liver disease is characterized by hepatocyte apoptosis, necrosis, and senescence (Ghavami et al., 2005), and is concomitantly associated with a robust expansion of the LPC compartment (Duncan et al., 2009; Fellous et al., 2009). Some of the putative factors that promote LPC expansion include cytokines IL6, TNFα, TGFβ, as well as cytokine regulated transcription factors nuclear factor kappa B, CCAAT enhancer binding protein beta, and growth factors HGF, EGF (Campbell et al., 2001). Hormones (such as insulin, somatostatin) (Jung et al., 2012), adipokines (cytokines released by adipocytes, such as leptin) (Diehl, 2005; Nobili et al., 2012), and neurotransmitters (such as serotonin, epinephrine or norepinephrine) have also been reported to regulate LPC response or growth. The interactions between these factors and signaling pathways are complex, and remain poorly understood. In aggregate, they act to stimulate the proliferation of LPC, and promote their differentiation into new hepatocytic cells and cholangiocytes. Recent studies show that morphogens (factors important during embryonic development) such as Wnt, Notch, and Hedgehog (Hh) are also important drivers of LPC response. For example, Hh ligands released by apoptotic hepatocytes can act on surrounding LPC and hepatic stellate cells (the key cell involved in scar tissue accumulation) to promote liver repair (Jung et al., 2010), while Wnt and Notch signals within the microenvironment could modulate LPC differentiation into either hepatocytes or cholangiocytes, respectively (Boulter et al., 2012).

The regenerative process that follows ALF is not well described or understood, but is likely to resemble the liver repair process occurring during chronic liver disease. During ALF, the liver would have been subject to a significant insult that results in widespread hepatocyte necrosis and apoptosis, which far exceeds the capacity of the remaining healthy hepatocytes to replicate and to restore homeostatic function.

For individuals who have other co-morbidities (such as hepatic steatosis associated with obesity or type 2 diabetes mellitus), hepatocytes may already exhibit replicative senescence which would further limit the regenerative capability of residual hepatocytes. The LPC compartment located within the canals of Herring (Petersen and Shupe, 2008) is therefore tasked to restore hepatocytic function in the failing liver. Indeed, in a recent study (Dechene et al., 2010), we observed a robust ductular reaction among survivors of ALF. Consistently, LPC markers appeared to correlate with severity and short-term mortality among individuals with alcoholic hepatitis (Sancho-Bru et al., 2012). Following fulminant liver injury, several different cell signaling axes have also been postulated to regulate LPC-mediated liver regeneration.

The TNF-like weak apoptosis inducing factor (TWEAK)/Fibroblast growth factor inducible 14 (Fn14) pathway plays a crucial role in activation of LPCs. TWEAK/Fn 14 activation has been reported to selectively expand LPCs, without affecting growth and viability of mature resident hepatocytes (Jakubowski et al., 2005). Recent studies in humans and mice with chronic liver disease confirmed that Fn14, the receptor of TWEAK, is dramatically upregulated during chronic injury, and directly modulates the LPC response (Tirnitz-Parker et al., 2010). Liver expression of TWEAK/Fn14 is also upregulated significantly, early after partial hepatectomy (Ochoa et al., 2010). Using the Fn14-deficient mice, Karaca and colleagues further propose that TWEAK/Fn14 axis could directly stimulate LPC expansion after acute liver injury (Karaca et al., 2010).

Following activation of progenitor cell niche, it has been postulated, that migration of LPCs is mediated by SCF/c-Kit (Hu and Colletti, 2008) and SDF1/CXCR4 (Hatch et al., 2002):

Stem cell factor (SCF) and its receptor c-kit play a key role in hematopoiesis and cellular proliferation. It is well accepted, that c-kit is a cell surface marker for progenitor cells (Heinemann et al., 2005). The biologic effects of the SCF/c-kit system are believed to involve survival, proliferation, and migration of early stem cell progeny (Fujio et al., 1994). There is a large reservoir of hepatic SCF, and this molecule has proven to play a pivotal role in liver reconstitution following 70% partial hepatectomy in mice. In another mouse model of ALF, Hu and co-workers found SCF-deficient mice administered APAP exhibited significantly higher mortality compared with litter-mate controls. Furthermore, administration of exogenous SCF significantly reduced mortality of APAP-treated wild-type mice (Hu and Colletti, 2008).

Stromal derived factor-1 alpha (SDF-1alpha) and its cognate receptor CXCR4 have similarly been shown to regulate migration of hematopoietic stem cells (HSC) in the fetal and adult stages of hematopoiesis. Previously, others have shown that bone marrow-derived mesenchymal stem cells promote hepatic regeneration *after CCl 4 treatment in rats* (Gruttadauria et al., 2013; Li et al., 2013). Hatch and colleagues recently proposed that the SDF-1alpha-CXCR4 axis is important for oval (LPC) cell activation during liver regeneration. They show that up-regulation of hepatocyte-derived SDF-1alpha expression during fulminant liver injury could not only promote recruitment of HSC from the bone-marrow (Hatch et al., 2002), but also enhance LPC accumulation, as both of these progenitor populations express CXCR4, the known receptor for SDF-1alpha.

The Hh pathway normally orchestrates fetal tissue and organ development, but has been shown to play an important role during adult tissue repair (Omenetti and Diehl, 2008). Hh ligands stimulate the expansion and viability of various stem cells (Yang et al., 2008b) and have been shown to function as viability factors for human and rodent liver progenitors. Recent studies have shown that Hh pathway activation occurs during liver regeneration after partial hepatectomy (Ochoa et al., 2010; Cai et al., 2011; Hanaoka et al., 2013). Importantly, inhibiting the Hh signaling (with cyclopamine, an antagonist of Smoothened), led to an attenuated LPC response, a lower expression of liver progenitor markers, AFP, Fn14, and K19, and reduced overall survival (Ochoa et al., 2010). The aggregate observations confirm the importance of Hh signaling in the LPC response after acute liver injury, and suggest that the degree of Hh pathway activation may dictate the extent of liver regeneration and clinical outcome in ALF. Although consistent with its recognized role during development, further studies will be needed to ascertain the importance of Hh-mediated LPC response in other models of adult ALF. A better understanding of the role of LPC in ALF, and more detailed study of some of these pathways may help identify potential treatment strategies for the treatment of ALF.

The role of Tri-iodothyronine (T3) in tissue regeneration is well recognized (Leffert and Alexander, 1976; Short and Ove, 1983). Experimental models of liver regeneration have, in most cases, focused on characterizing hepatocyte replication, but not of LPC-mediated parenchymal reconstitution. T3 affects cell growth, differentiation, and regulates metabolic functions via its interaction with the thyroid hormone nuclear receptors (TRs). Cumulative studies suggest that T3 is a potent stimulator of liver regeneration. Bockhorn et al. found out that the exogenous administration of T3 enhanced liver regeneration after 70 and 90% hepatectomy in terms of increased liver to body weight ratio and Ki-67 index (Bockhorn et al., 2007). Several molecular mechanisms have been postulated to mediate T3 effects on liver regeneration. T3 stimulated rats that were subjected to partial hepatectomy expressed the cell cycle protein, cyclin D1 at earlier time points compared with control rats that did not receive T3, suggesting that T3-TR signaling is an important regulator of the cell cycle in an experimental model of liver resection (Leffert and Alexander, 1976; Short and Ove, 1983). Recent reports suggest that T3 not only stimulates hepatocyte proliferation, but may induce LPC activation during fulminant liver injury. In a rodent model of combined AAF/PH, László et al. showed that administration of T3 led to an accelerated differentiation of LPCs into hepatocytes (Laszlo et al., 2008). Nevertheless, the molecular mechanisms underlying the LPC differentiation response have yet to be fully understood.

## Is there a cellular micro-environment chaperoning LPC migration?

**Figure 1:**
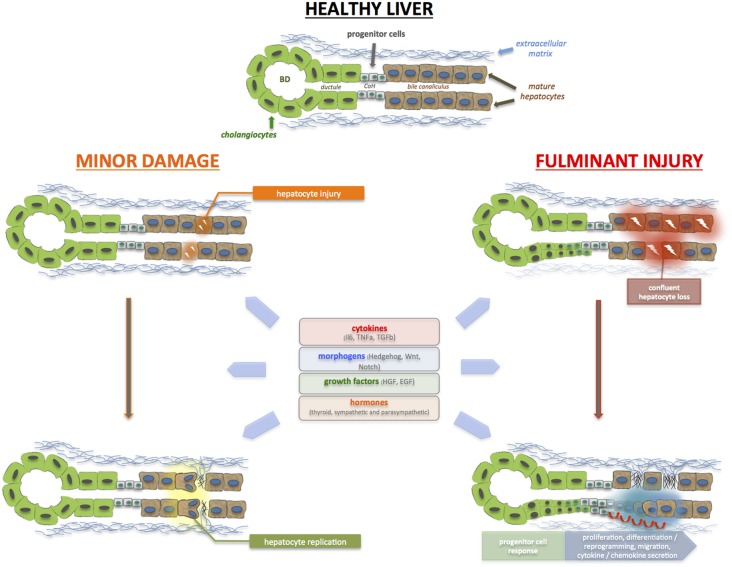
**During acute liver injury, hepatocyte (and cholangiocyte) apoptosis, and necrosis occur.** With minor injury, restoration of hepatocyte mass and function is mediated by the replication of remaining healthy hepatocytes (and cholangiocytes). During a major insult, massive, and confluent hepatocyte loss occurs. There are insufficient healthy remaining hepatocytes mass to restore hepatic function; as such, the liver progenitor (LPC) or liver stem cell compartment is activated in an attempt to restore epithelial cell mass, architecture, and function. The bipotential LPCs reside in the Canals of Herring (CoH), located in the niche of the biliary-hepatocytic interface, and are able to infiltrate along the liver plate and differentiate into hepatocytes and cholangiocytes. LPCs are surrounded by epithelial cells, non-parenchymal cells such as hepatic stellate cells, as well as immune cells and extracellular matrix. These regenerative processes are triggered and regulated by the plethora of cytokines, growth factors and metabolic signals. Resurrection of morphogenic signals (i.e., Hedgehog, Wnt, Notch) also occurs, particularly during massive injury, to invoke the liver progenitor cell compartment. In brief, these molecules act in concert to ensure that sufficient regeneration occurs, and yet, not exceed normal homeostatic requirements.

LPCs are believed to originate from the canals of Hering (CoH), which is lined proportionately by cholangiocytes and hepatocytes (see Figure 1). It serves to conduct bile from the bile canaliculi to the terminal bile ducts located in the portal tracts (Saxena and Theise, 2004). Because the CoH constitutes the biliary-hepatocytic interface, it makes biological sense that LPCs, being bipotential cells, are located in this niche. The LPC neighborhood includes epithelial (hepatocytes and cholangiocytes) cells, hepatic stellate cells, immune cells (i.e., Kupffer cells), and the extracellular matrix (ECM). The proximity of these cells suggests that crosstalk is important, and occurs not only under basal, homeostatic conditions, but also during injury and repair. Indeed, during liver injury, soluble factors released by one cell type act in a autocrine and paracrine manner to regulate the growth and differentiation of a neighboring cell compartment (Parola and Pinzani, 2009). We reported that Hh ligands which are over-expressed during acute and chronic liver injury could directly stimulate LPCs to secrete chemokines that lead to the additional recruitment of inflammatory cells which participate in the regeneration or repair process (Omenetti et al., 2009). Inflammatory cells produce a range of cytokines and chemokines. SDF-1 attracts CXCR4^+^ T cells, which express TNF-like weak inducer of apoptosis (TWEAK), that in turn stimulates LPC response by engaging its receptor Fn14 (Alison et al., 2009).

Recent studies further show that the expansion of the LPC compartment occurs in association with ECM remodeling (Van Hul et al., 2009; Lorenzini et al., 2010; Lozoya et al., 2011), while failure of ECM remodeling lead to impaired ability of the liver to activate LPCs (Kallis et al., 2011). In this study, laminin-LPC interactions were shown to be critical for LPC-mediated repair. Separately, Van Hul and co-workers observed that ECM deposition and activation of matrix-secreting cells occurred, not only before the increase in number of LPCs, but also in front of LPCs along the porto-venous gradient of lobular invasion (Van Hul et al., 2009). During migration, LPCs are embedded within ECM and are chaperoned by alpha-smooth muscle actin (alpha-SMA)-positive cells (Van Hul et al., 2009). In addition to a direct effect of Hh on LPC, Hh pathway activation could also enhance LPC proliferation, indirectly, through the activation of hepatic stellate cells into matrix-producing myofibroblasts (Choi et al., 2009). These findings in mice support our study in man, where we showed that LPC expansion (ductular reaction) occurred in context of the fibrogenic response (Dechene et al., 2010). We propose that short term accumulation of collagen matrix might be a physiological repair response that precedes parenchymal cell reconstitution. Similar to findings in mice, the fibrous tissue scaffold is the LPC niche that facilitates LPC activation and differentiation.

Further studies will be needed to understand if, and how ECM composition could modulate LPC responses after ALF, and whether modifying individual components of ECM could regulate LPC proliferation and differentiation.

## Is there a correlation between LPC activation and clinical parameters in alf?

Currently used ALF scoring systems such as the KCC, MELD, and BiLE reliably predict death, but are poor at predicting survival (Polson and Lee, 2005). None of these systems take into account the histological changes that occur during ALF. The role of histology and presence or absence of hepatocyte cell death has only recently come to fore. We recently reported that a CK18 M65 (marker of cell death)-based MELD score could predict survival from ALF with greater sensitivity and specificity (Bechmann et al., 2010). Among individuals with acute alcoholic hepatitis, a condition with high mortality, expression of LPC markers correlated positively with clinical severity and short-term mortality (Sancho-Bru et al., 2012).

A similar study of 74 patients with ALF or sub-acute liver failure demonstrated a positive correlation between histopathological findings (of hepatocyte loss, the number of proliferating hepatocytes and the number of LPCs) and clinical severity by the MELD score. The fact that the number of LPCs was not relevantly different between mild (30%) and moderate (30–50%) hepatocyte loss, but significantly increased upon severe (50–75%) or very severe (>75%) injury, clearly indicates that LPC compartment activation requires a high parenchymal injury threshold prior to its recruitment (Katoonizadeh et al., 2006). Importantly, surviving patients exhibited significantly fewer hepatocyte losses, less LPC activation and more mature hepatocyte proliferative activity, compared with those who either died or needed a liver transplant.

The cumulative data show that the degree of LPC activation and expansion of LPC compartment correlates strongly with the extent of hepatic injury and severity of ALF. Further studies will certainly be needed to evaluate if liver histology would improve prognostication (or predicting survival) for patients with ALF.

## Could LPCs represent a future cell therapy option in alf?

Whether the LPC response is simply a bystander effect as a result of the rich cytokine milieu, or whether it is an incomplete or unsuccessful attempt at liver regeneration remains to be seen. However, the significance of LPC activation during recovery from acute liver injury remains subject of controversy. Lineage tracing models utilizing reporter mouse models might represent a feasible tool to quantify the contribution of LPC during regeneration (Malato et al., 2011; Espanol-Suner et al., 2012; Diehl and Chute, 2013).

At present, the only curative treatment for patients with fulminant hepatic failure is an ELT. However, this is significantly limited by the shortage of suitable donor organs. As such, hepatocyte (cell) transplantation has been evaluated as an alternative for those ineligible for liver transplantation, or as a bridge to liver transplant. This is particularly attractive because cryopreserved cells are readily available. However, the number of cells that can be delivered (via the portal vein) is limited by the risks of portal hypertension (Weber et al., 2009a, b), and their large size lead to reduced cellular engraftment (Fox et al., 1998). LPC, on the other hand, are small in size, and are capable of differentiating into both hepatocytes and cholangiocytes (Sandhu et al., 2001). It would be important to study if enhanced expansion of the LPC compartment, with or without changes in the LPC niche, could lead to amelioration of ALF.

Previously, the limiting factor in the study of LPC has been the inability to identify, isolate or purify these cells in a reliable fashion. Recently, Cardinale and colleagues successfully isolated multipotent stem/progenitor cells from the human biliary tree by extended cell culture techniques (Cardinale et al., 2011) and demonstrated that these progenitor cells are capable of giving rise to hepatocytes, cholangiocytes, and pancreatic islets. We have similarly developed a LPC isolation protocol for mouse and human liver tissue, but using fluorescence-activated cell sorting (FACS). Our technique was based on the observation that progenitor cells express high levels of aldehyde dehydrogenase (ALDH) activity. FACS-ALDH positive LPC in culture could give rise to functional hepatocyte-like cells as illustrated by albumin and urea secretion and cytochrome P450 activity. These novel methods of LPC isolation could well pave the way for the development of future ALF therapies (Dollé et al., 2012).

A non-parenchymal cell population that might have an implication in liver regeneration are hepatic stellate cells. Their contribution to liver regeneration was recently confirmed with pancreatic stellate cells that were transplanted via tail vein injection into rats that were previously subjected to 70% PH and substantially contributed to organ reconstitution by differentiating into epithelial cells. The contribution of stem cells in tissue repair remains controversial, but prevailing evidence suggest that bone marrow or adipose tissue derived MSCs might contribute to liver regeneration through differentiation (Sato et al., 2005; Aurich et al., 2007; Chamberlain et al., 2007). Hepatic stellate cells could possibly fulfill a dual role as supportive cells producing a connective tissue scaffold facilitating LPC expansion and migration on the one hand and as progenitor cells on the other (Yang et al., 2008a; Kordes and Haussinger, 2013).

The inter-relationship between liver and non-liver progenitor or stem cells (i.e., bone marrow derived), and their roles in liver regeneration after ALF remains complex and poorly understood. Further studies will be necessary to understand and tap this potential source of new liver cells. Administration of granulocyte colony stimulating factor (G-CSF) during myocardial infarction for example, leads to the mobilization and differentiation of HSC to a committed lineage (Theiss et al., 2013). Therefore, a potential attempt to enhance liver regeneration during ALF might be a mobilization of bone marrow progenitor cells by an administration of G-CSF.

## Conclusions

Further studies will be needed to understand the role of the LPC response during ALF. Cumulative data to date suggest that the LPC compartment is activated when there is confluent loss of hepatocyte mass, that lead to insufficient regenerative capacity of residual hepatocytes. The cytokine storm that ensues during acute liver injury, in combination with growth factors, morphogens, hormones, and neurotransmitters, all act in concert to dictate the LPC response. LPC activation and differentiation appears to require ECM, and forms the LPC niche. Hence, modulating the ECM composition and/or enhancing the LPC response could be useful strategies to promote liver regeneration.

Observational studies from cohorts of patients with ALF show that the amount and type of LPC activation/expansion correlate with severity of liver injury, and clinical outcomes. This is unsurprising as hepatocyte cell death is intricately associated with liver repair (i.e., the greater the injury, the greater the attempt at repair). Currently used scoring systems have not been able to reliably predict those who may survive from ALF. Future studies will be needed to evaluate if the degree of LPC response and/or ECM accumulation could be useful biomarkers of regenerative capability, thus improving clinical decision making in ALF.

## Funding

Foundation for Liver Research (Wing-Kin Syn, Jason Coombes), CORE (Wing-Kin Syn), BRET (Wing-Kin Syn), Belgian Federal Science Policy Office (Interuniversity Attraction Poles program - P6/20 and P7/83-HEPRO) (Jan Best, Laurent Dollé, and Leo A. van Grunsven), the Brussels Capital Region (INNOVIRIS Impulse programme-Life Sciences 2007 and 2011; BruStem) (Jan Best, Laurent Dollé, and Leo A. van Grunsven) and the Institute for the Promotion of Innovation through Science and Technology in Flanders (SBO-IWT-090066 HEPSTEM) (Jan Best, Laurent Dollé, and Leo A. van Grunsven).

### Conflict of interest statement

The authors declare that the research was conducted in the absence of any commercial or financial relationships that could be construed as a potential conflict of interest.
